# Monitoring Membrane Fouling Using Fluid Dynamic Gauging: Influence of Feed Characteristics and Operating Conditions

**DOI:** 10.3390/membranes13100834

**Published:** 2023-10-19

**Authors:** Kenneth Arandia, Nabin Kumar Karna, Tuve Mattsson, Hans Theliander

**Affiliations:** 1 Department of Chemistry and Chemical Engineering, Chalmers University of Technology, SE-412 96 Gothenburg, Sweden; nabin@chalmers.se (N.K.K.); hanst@chalmers.se (H.T.); 2 Wallenberg Wood Science Center, Chalmers University of Technology, SE-412 96 Gothenburg, Sweden; 3 Center for Membrane Technology, Department of Chemistry and Bioscience, Aalborg University, Fredrik Bajers Vej 7H, DK-9220 Aalborg, Denmark; tuvem@bio.aau.dk

**Keywords:** membrane fouling, fluid dynamic gauging, microcrystalline cellulose, microfiltration

## Abstract

Recent studies on membrane fouling have made considerable progress in reducing its adverse effects. However, a lack of comprehensive studies focusing on the underlying fouling mechanisms remains. This work aims to address a part of this gap by investigating the influence of feed suspension chemistry and operating conditions on the fouling characteristics of microcrystalline cellulose. Fluid dynamic gauging (FDG) was employed to monitor the properties of fouling layers under varied conditions. FDG results revealed that the cohesive strength of fouling layers increased in the direction towards the membrane, which can be associated with the higher compressive pressures exerted on foulants deposited near the surface. At lower pHs and higher ionic strengths, reduced electrostatic repulsions between particles likely resulted in particle agglomeration, leading to the formation of thicker cakes. In addition, thicker cake layers were also observed at higher feed concentrations, higher operating transmembrane pressures, and longer filtration times. The cross-flow velocity influenced the resilience of fouling layers significantly, resulting in thinner yet stronger cake layers in the transition and turbulent flow regimes. These findings regarding the influence of feed characteristics and operating conditions on the fouling behavior can be beneficial in developing effective antifouling strategies in membrane separation processes.

## 1. Introduction

Membrane technology has thrived over the years as a separation process that offers a more energy-efficient and cost-effective pathway than conventional thermal separation methods such as evaporation, crystallization, and distillation. Pressure-driven membrane processes, which include microfiltration (MF), ultrafiltration (UF), nanofiltration (NF), and reverse osmosis (RO), are employed in a wide range of applications (e.g., food and beverage production, pharmaceutical technology, and wastewater treatment). Despite the technological advancements that have been made in membrane separation, the persistent challenge that still limits its performance is fouling, i.e., the deposition of dissolved and suspended substances on the surface or within the pores of a membrane [1]. The decline in performance due to fouling entails additional operational and maintenance costs to cover cleaning procedures and changing out membranes. An in-depth knowledge of the fouling mechanisms is therefore necessary to develop appropriate antifouling strategies.

In biorefineries and pulping processes, the initial step when utilizing lignocellulosic biomass, such as wood, involves decoupling its various constituents: cellulose, hemicelluloses, and lignin. Their subsequent separation can be accomplished using membrane processes [2]; however, these wood constituents can potentially foul the membrane systems employed in the fractionation and concentration of the desired components. Studies on pressure-driven membrane filtration have been conducted using process waters from thermomechanical pulp mills [3,4,5], Kraft black liquor [6,7,8], and spent sulfite liquor [9,10,11]. These studies highlight the complex interactions of wood components, necessitating advanced techniques for monitoring their fouling behavior rather than relying solely on changes in permeate flux and transmembrane pressure (TMP). It is essential to employ in situ and real-time techniques to monitor the build-up of fouling layers in order to gain insights into the fouling characteristics of wood components during membrane separation.

Out of the in situ and real-time techniques available for investigating membrane fouling, fluid dynamic gauging (FDG) offers valuable insights into the fouling characteristics by estimating the thickness and strength of fouling layers formed on a membrane surface. It is simple, inexpensive, and versatile, with automated features for time-efficient monitoring of the build-up of fouling layers. Tuladhar et al. developed FDG initially as a proximity gauging technique for soft deposits on stainless steel surfaces [12]. Since then, its application has been extended to investigate membrane fouling caused by various foulants, such as glass ballotini suspensions [13,14,15], sugar beet molasses [16], yeast suspensions [17], titanium dioxide (TiO${}_{2}$) particles [18], biofilms [19], orange juice [20], and microcrystalline cellulose (MCC) [21,22,23].

MCC is a purified and partially depolymerized cellulose produced through the hydrolysis of delignified wood fibers using a strong acid. It is a valuable additive in the food, pharmaceutical, cosmetic, and polymer composites industries, functioning as a binder, emulsifier, stabilizer, and bulking agent [24]. The extensive range of MCC applications stems primarily from its favorable properties, which include a high specific surface area, high mechanical strength and stiffness, low density, biodegradability, biocompatibility, and renewability. However, its highly hygroscopic nature, poor solubility, incompatibility with most polymeric matrices, and high specific filtration resistance in solid–liquid separation restrict its processability, thus necessitating further research.

Several studies have been carried out on the dead-end filtration of MCC to examine the influence of feed characteristics and process conditions on filtration properties. These investigations involved modifying parameters such as ionic strength [25], pH and specific surface area [26] of the feed suspensions. For cross-flow filtration, the influence of pH [21] and cross-flow velocity (CFV) [22] have been examined. These studies reported that thicker and stronger fouling layers were formed, and a slower decline in permeate flux occurred at an acidic pH than at close-to-neutral pH. Moreover, under transitional/turbulent flow conditions, more resilient fouling layers were formed than those formed under laminar conditions. Despite the significant insights gained from these studies regarding the filtration properties of MCC, a comprehensive parametric analysis for cross-flow MF has yet to be conducted.

The filtration properties provide relevant information on the different filtration behavior of a material under varied conditions. In this work, the fouling characteristics of MCC during cross-flow MF are investigated using FDG by altering the feed suspension chemistry (pH, ionic strength, and feed concentration) and operating conditions (TMP, filtration time, and CFV). The focus is placed on determining the individual effects of these key parameters on the thickness and strength of the fouling layers formed on flat-sheet polyethersulfone (PES) membranes under various conditions.

## 2. Materials and Methods

### 2.1. Fluid Dynamic Gauging

**Figure 1:**
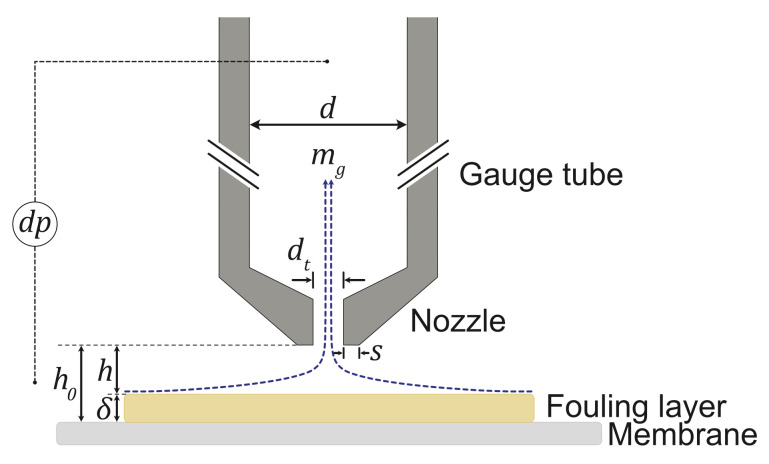
Schematic diagram of an FDG probe approaching the surface of a fouling layer formed on a flat-sheet membrane. *h*${}_{0}$ is clearing height over the membrane surface, *h* is the clearing height over the fouling layer, δ is the thickness of the fouling layer, *dp* is the pressure drop over the FDG probe, *m*${}_{g}$ is the mass flow rate through the gauge, *d* is the inner diameter of the gauge tube (3 mm), *d*${}_{t}$ is the inner diameter of the nozzle (0.5 mm), and *s* is the radial width of the gauge nozzle rim (0.25 mm).

Fluid dynamic gauging (FDG) is an in situ and real-time technique for monitoring the deposition of foulants on solid surfaces. In pressure-driven membrane processes, it allows the thickness and strength of the fouling layers formed on porous membrane substrates to be estimated. A physical probe, comprising a gauge tube and a tapered nozzle, is employed to approach the surface of a fouling layer while fluid is drawn through the probe. This action generates a pressure drop caused by the flow being constricted between the tip of the nozzle and the surface of the fouling layer [13], as illustrated in Figure 1.

There are two modes of operation for conducting FDG measurements: (i) the mass flow mode, where the pressure drop over the FDG probe, *dp*, is kept constant, and (ii) the pressure mode, where the mass flow rate through the gauge, *m*${}_{g}$, is maintained constant. This study uses pressure-mode FDG, with the measurements being taken while fluid was withdrawn at *m*${}_{g}$ = 0.1 g s${}^{-1}$. At a known membrane position or clearing height over the membrane surface, *h*${}_{0}$, a correlation between the *dp* values and the clearing height over the fouling layer, *h*, can be calculated. The thickness of the fouling layer, δ, can then be estimated using Equation (1):

(1) $$\delta =h_{0}-h$$

where *h*${}_{0}$ and *h* are the probe clearing heights over the membrane and the fouling layer, respectively.

As the FDG probe is continuously lowered to approach the fouling layer, the fluid flow through the gauge imposes a tangential shear stress upon the surface of the fouling layer. This allows the successive removal of cake layers to be monitored due to the increased applied fluid shear. The shear-induced removal of individual cake layers enables the local cohesive or adhesive strength at different positions in the fouling layer to be estimated. The region directly beneath the inner edge of the nozzle rim, at a radial distance *d*${}_{t}$/2 from the centerline, experiences the largest shear stress, $\tau_{w,\max}$. The value of $\tau_{w,\max}$ can be calculated using Equation (2), assuming a creeping concentric flow between the parallel plates:

(2) $$\tau_{w,\max}=\frac{6\mu m_{g}}{\pi \rho h^{2}}\cdot \frac{1}{d_{t}}$$

where μ and ρ are the dynamic viscosity and density of the fluid, $m_{g}$ is the mass flow rate through the gauge, *h* is the clearing height over the fouling layer, and *d*${}_{t}$ is the inner diameter of the nozzle.

The linear form of Darcy’s equation for fluid flow through a porous medium can be employed to calculate the permeability of the cake, as indicated by Equation (3):

(3) $$Q=\frac{\mathrm{kA}}{\mu L}\Delta p=-\frac{\mathrm{kA}}{\mu }\nabla p=-\frac{\mathrm{kA}}{\mu \delta }\mathrm{TMP}$$

where *Q* is the volumetric flow rate [m${}^{3}$ s${}^{-1}$], *k* is the permeability of the cake [m${}^{2}$], *A* is the cross-sectional area [m${}^{2}$], Δ*p* is the total pressure drop [N m${}^{-2}$], μ is the dynamic viscosity [N m${}^{-2}$ s], *L* is the length of the sample [m], ∇*p* is the hydraulic gradient applied between points a and b [N m${}^{-1}$], TMP is the transmembrane pressure [N m${}^{-2}$], and δ is the thickness of the cake [m].

### 2.2. Cross-Flow Filtration Equipment

**Figure 2:**
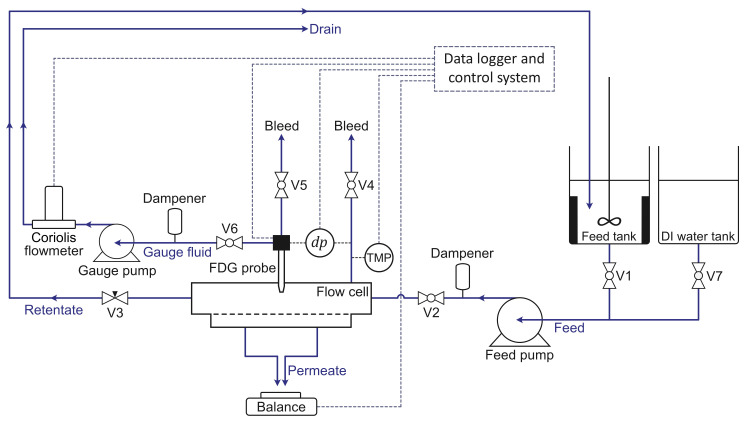
Schematic diagram of the cross-flow filtration equipment in a *feed-and-bleed* configuration, depicting the feed, permeate, retentate, and gauge fluid streams.

Figure 2 is a schematic diagram of the bench-scale, stainless-steel filtration test rig used in the cross-flow MF experiments. It comprised the following components: a flow cell with dimensions of 150 mm × 16 mm × 15 mm (L × W × H), an FDG probe positioned at the center of the flow channel, a precision balance for permeate collection, a baffled feed tank and a gear pump for circulating the feed suspensions, a deionized water tank, a gear pump and a Coriolis flowmeter for gauging flow control, two pressure transducers for *dp* and TMP measurements, two pressure fluctuation dampeners, a needle valve (V3) for TMP regulation, and six ball valves, two of which were bleed valves. Detailed technical specifications of each component can be found elsewhere [27]. Key elements of the test rig were linked to a data logger and control system and their output data monitored via LabVIEW^TM^ 2020 (National Instruments, Austin, TX, USA).

### 2.3. Materials

Commercially available microcrystalline cellulose (Avicel^®^ PH-105 MCC, DuPont Nutrition, Little Island, Cork, Ireland), with a nominal particle size of 20 μm, was selected as the model foulant in this study. The same batch of MCC was used in prior investigation, wherein its solid density and specific surface area were determined to be 1560 kg m${}^{-3}$ and 39 m${}^{2}$ g${}^{-1}$, respectively [25].

Hydrophilic polyethersulfone membranes (Supor^®^ PES, Pall Corporation, Ann Arbor, MI, USA), with a nominal pore size of 0.45 μm, were used in all cross-flow MF experiments. The PES membranes exhibit a pure water flux of 58 mL cm${}^{-2}$ min${}^{-1}$ (3.48 × 10${}^{4}$ L m${}^{-2}$ h${}^{-1}$) at 0.7 bar TMP, as specified by the manufacturer.

Sulfuric acid (95–97% H${}_{2}$SO${}_{4}$, Merck, Darmstadt, Germany), sodium hydroxide (EMPLURA^®^ ≥ 98% NaOH, Merck, Darmstadt, Germany), and sodium chloride (NaCl, Avantor, Stockholm, Sweden) were added to modify the pH and ionic strength of the MCC suspensions.

### 2.4. Preparation of the Feed Suspensions

An MCC suspension, with a concentration of 0.15 vol%, was prepared for each filtration experiment by suspending MCC particles in deionized water. Mechanical pretreatment of the suspension was performed for 15 min using a dispersing instrument (IKA T50 digital Ultra-Turrax^®^, IKA-Werke GmbH, Staufen, Germany) operated at 10,000 rpm, with an S50 N-G45F dispersing element. Subsequently, the suspension was transferred to a 5 L baffled vessel and stirred continuously at 300 rpm, using a pitched two-blade impeller for a minimum of 12 h, to ensure consistent swelling of the particles.

The mechanically treated 0.15 vol% suspension was diluted further to prepare feed suspensions of different concentrations: 0.01, 0.02, and 0.04 vol%. The influence of all parameters, with the exception of feed concentration, was tested at a constant volume fraction of 0.02 vol%. MCC suspensions were prepared at three pH levels (4, 6, and 9) and three ionic concentrations (1, 2, and 3 mM NaCl). The pH of the feed suspensions was adjusted using 1 M H${}_{2}$SO${}_{4}$ or 1 M NaOH, whilst the ionic strength was modified using a 2 M NaCl stock solution. The feed suspensions were placed in a baffled feed tank under continuous stirring, at an ambient temperature of 22–23 °C.

### 2.5. Membrane Filtration Experiments

Prior to each cross-flow MF experiment, a PES membrane was cut into a rectangular sheet (200 mm × 30 mm) and initially rinsed, via soaking in deionized water, for at least half an hour. The membrane was then mounted into the flow cell, employing two support layers comprising a porous polypropylene sheet and a perforated stainless-steel slab with 2 mm holes. The active membrane surface area measured 2.4 × 10${}^{-3}$ m${}^{2}$ (150 mm × 16 mm).

An initial suspension volume of either 5 or 10 L was used for the cross-flow MF experiments, in which the starting feed volume was based on the permeate flux values. Suspensions with a 10 L capacity were prepared at pH 4 and higher ionic concentrations (2 and 3 mM) to prevent complete depletion of the feed due to relatively high permeate flux values. For the tests carried out under various operating conditions, the following were investigated, namely three TMPs: 200, 300, and 400 mbar (±5%); three filtration times: 10, 30, and 50 min; and three CFVs: 0.10, 0.18, and 0.30 m s${}^{-1}$. These CFVs correspond to a duct flow Reynolds number, *Re*${}_{\mathrm{duct}}$, of 1700, 3100, and 4900, respectively.

**Figure 3:**
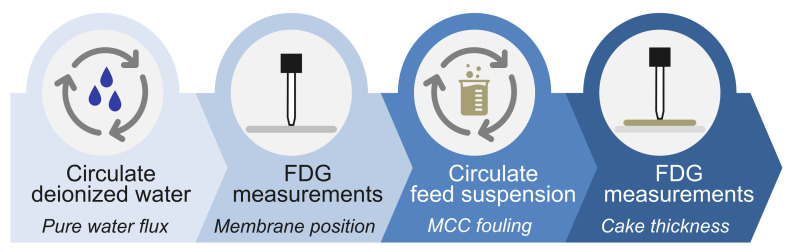
Process flow of the cross-flow MF of the MCC suspensions and FDG measurements.

The process flow of the cross-flow MF of the MCC suspensions is illustrated in Figure 3. Three sets of MF experiments were conducted for each parameter. Each MF experiment comprised the following steps: circulation of deionized water to verify the pure water flux, FDG measurements to determine the position of the membrane, MCC fouling through the circulation of the feed suspension for a specified filtration time, and, finally, FDG measurements to estimate the thickness and strength of fouling layers.

A volume of 5 L of deionized water was circulated throughout the filtration test rig for at least 30 min. The pH or ionic strength of the deionized water was adjusted during this conditioning step according to the specific parameter being investigated. The pure water flux was calculated using Equation (4):

(4) $$J=\frac{\Delta m}{\Delta t}\cdot \frac{1}{\rho A}$$

where *J* is the flux [L m${}^{-2}$ h${}^{-1}$], Δ*m*/Δ*t* is the mass flow, ρ is the density of the fluid, and *A* is the active membrane surface area (2.4 × 10${}^{-3}$ m${}^{2}$).

The position of the membrane was determined via FDG measurements by moving the probe towards the membrane surface incrementally, while simultaneously withdrawing deionized water at a constant gauging mass flow of *m*${}_{g}$ = 0.1 g s${}^{-1}$. The *dp* and *h*${}_{0}$/*d*${}_{t}$ values were recorded, and the measurements were carried out until the *dp* reached 100 mbar, i.e., the maximum *dp* set to prevent any potential damage to the membrane by the probe. A damaged membrane could affect the flux measurements and the deposition of foulants due its surface structures being altered. To confirm its position, the membrane calibration curve (*dp* vs. *h*${}_{0}$/*d*${}_{t}$) was shifted by superimposing it with the master calibration curve derived from FDG calibration. The FDG calibration data were taken from a previous study [23], in which the master calibration curve followed Equation (5):

(5) $$\mathrm{dp}=c_{1}{\left(\frac{h}{d_{t}}\right)}^{c_{2}}+c_{3}$$

where *c*${}_{1}$ = 0.098, *c*${}_{2}$ = −2.267, *c*${}_{3}$ = 4.703, and *R*${}^{2}$ = 0.999.

After determining the position of the membrane, the probe was retracted to the top of the flow cell to minimize its impact on the build-up of the fouling layer, and the gauge flow was stopped. The feed line was then redirected from deionized water to the MCC suspension, before the suspension was circulated for a specified filtration time: 10, 30, or 50 min, depending on the parameter under investigation. The permeate flux was also calculated, using Equation (4).

The gauge flow was reactivated after the specified filtration time had elapsed. Subsequent FDG measurements were made until *dp* reached 100 mbar. A plot of *dp* vs. *h*/*d*${}_{t}$ was generated to analyze the fouling layer build-up during cross-flow MF. The height of the fouling layer, δ, was determined by subtracting the measured *h*/*d*${}_{t}$ values from the calculated *h*${}_{0}$/*d*${}_{t}$ values obtained from the membrane calibration, as described in Equation (1). Thickness measurements were taken from a probe distance at which the fouling layer could withstand the minimum applied shear stress of approximately 34 Pa. Once all measurements were complete, the probe was retracted and the circulation of the MCC suspension was terminated. The filtration test rig was cleaned by circulating deionized water for at least an hour to eliminate residual foulants within the system.

### 2.6. Characterization Techniques

#### 2.6.1. Laser Diffraction

The particle size distribution of the MCC suspensions was analyzed using a laser diffraction instrument (Mastersizer 2000, Malvern Panalytical, Malvern, Worcestershire, UK), with a detection range of 0.02–2000 μm. Representative samples of the MCC suspensions were analyzed at varying pH levels and ionic concentrations. Triplicate measurements were performed for each sample to determine the volume-based size distributions. The refractive indexes selected for the sample material and dispersant (water) were 1.53 [28] and 1.33, respectively.

#### 2.6.2. Focused Beam Reflectance Measurement (FBRM)

The chord length distribution of particles and agglomerates present in the MCC suspension was analyzed via Focused Beam Reflectance Measurement (FBRM^®^ G400, Mettler Toledo, Columbia, MD, USA), with a detection range of 1–1000 μm. The measurements were carried out in a 1 L baffled vessel, equipped with a pitched two-blade impeller, at a stirring rate of 250 rpm. The FBRM probe was positioned at an angle 45° relative to the direction of flow.

Two sets of FBRM measurements were performed: one at 0.02 vol% (feed concentration during cross-flow MF) and the other at 0.15 vol% (initial concentration after preparation). The pH and ionic strength were varied for each set of measurements. The initial pH of the suspension was adjusted to pH 4 by adding 1 M H${}_{2}$SO${}_{4}$, whereas another suspension was added with 1 M NaOH to achieve a pH of 9. For the ionic strength test, the NaCl concentration was altered stepwise by adding 1 mM NaCl until a concentration of 3 mM was reached. Chord length and particle count data points were recorded continuously at a sampling interval of 10 s.

#### 2.6.3. Zeta Potentials

The zeta potentials of MCC particles in the feed suspensions were measured using a Zetasizer Nano ZS (Malvern Panalytical, Malvern, Worcestershire, UK), with triplicate measurements being made for each sample. Representative samples of the MCC suspensions under various pH levels and ionic concentrations were centrifuged for 5 min at 4500 rpm (Heraeus Megafuge 40R, Thermo Scientific, Osterode, Germany) before the zeta potential measurements were conducted. Following centrifugation, the supernatant was collected and transferred to disposable folded capillary cells for analysis: this procedure prevents MCC particles from settling, as it could have a significant effect on the count rate during measurements.

## 3. Results and Discussion

### 3.1. Characterization of the Feed Suspensions

The volume-based size distributions and the zeta potentials of MCC particles in the feed suspensions, at a concentration of 0.02 vol% under varying pH and ionic concentrations, are reported in Table 1. The D${}_{50}$ value, which represents the size at which 50 vol% of the particles are contained, is 21.6–21.8 μm for all conditions. This value corresponds closely to the nominal particle size provided by the manufacturer. Negligible differences in particle and agglomerate sizes were observed, even with variations in electrostatic interactions caused by altering the pH and ionic strength. This trend is likely attributed to the disintegration of loose agglomerates by shear forces generated by the circulation pump. In addition, the size distributions remained relatively constant across different pH levels, even though cellulose tends to swell in water, with rapid swelling occurring under aqueous alkaline conditions [29]. Similar size distributions were also obtained at a feed concentration of 0.15 vol%, as shown in Table A1 in Appendix A, since particle sizing was performed on dilute suspensions.

**Figure A1:**
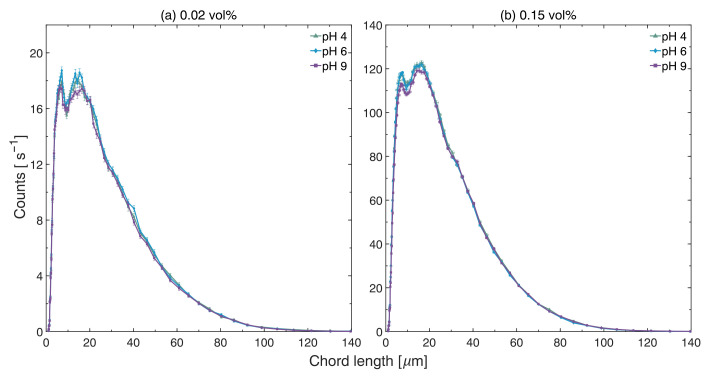
Chord length distributions obtained by FBRM for MCC suspensions at varying pH levels, with measurements conducted at feed concentrations of (**a**) 0.02 vol% and (**b**) 0.15 vol%. The pH was adjusted using 1 M H${}_{2}$SO${}_{4}$ or 1 M NaOH.

**Figure A2:**
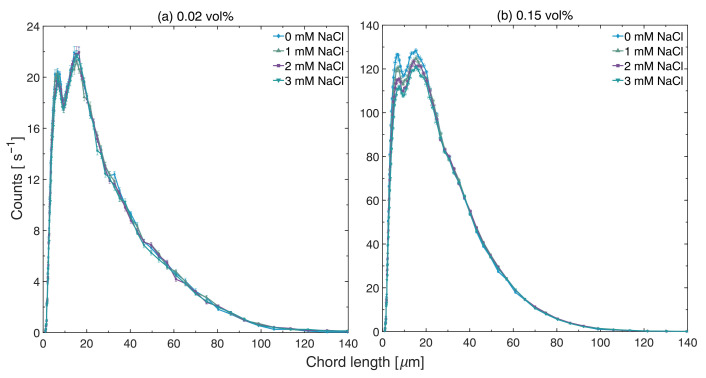
Chord length distributions obtained by FBRM for MCC suspensions at varying ionic strengths, with measurements conducted at feed concentrations of (**a**) 0.02 vol% and (**b**) 0.15 vol%. The NaCl concentration was changed stepwise by adding 1 mM NaCl.

The FBRM results, as presented in Figure A1 and Figure A2 in Appendix B, show no significant differences in the chord length distributions of the feed suspensions at 0.02 vol%, even when varying the pH and ionic concentration. At the higher feed concentration of 0.15 vol%, a subtle reduction in counts becomes apparent in the shorter chord lengths during the ionic strength test due to changes in the electrostatic interactions. In a previous work by Lidén et al. [25], the chord lengths shifted towards larger particle or agglomerate sizes with an increase in ionic concentration; they, however, worked with a substantially higher concentration of the MCC suspensions at 5 vol%. This observation indicates that changes in the chord lengths are more pronounced at higher concentrations as a result of more frequent interactions between particles.

The zeta potentials of MCC particles in the feed suspensions in Table 1 decreased in magnitude to −19.7 ± 2.2 mV at pH 4 from −33.4 ± 2.5 mV at pH 6. This decrease is plausibly attributed to weaker repulsive electrostatic forces, thereby promoting particle agglomeration. In contrast, the zeta potential increased marginally in magnitude from −33.4 ± 2.5 mV at pH 6 to −34.3 ± 3.0 mV at pH 9, as the increased repulsive electrostatic forces hindered the formation of agglomerates. Under neutral and alkaline conditions, MCC particles carry negative surface charges, ranging between −0.7 and −0.8 μeq g${}^{-1}$ [26]. Conversely, under acidic conditions, the particles exhibit reduced charge densities due to the protonation of carboxyl groups (p*K*${}_{a}$ = 3–4) [30]. Variations in electrostatic interactions were also observed at different ionic concentrations. The magnitude of the zeta potential decreased with increasing ionic strength, shifting from −33.4 ± 2.5 mV with no added ions (pH 6) to −13.5 ± 2.1 mV after adding 3 mM of NaCl. This response results from the compression of the electrostatic double layer, which enhances the shielding of negative charges at higher ionic concentrations, leading to the formation of larger agglomerates at a sufficiently high particle concentration. Regarding zeta potentials under varying feed concentrations, the values remained relatively constant, as shown in Table A2 in Appendix C.

### 3.2. Flux Profiles

**Figure A3:**
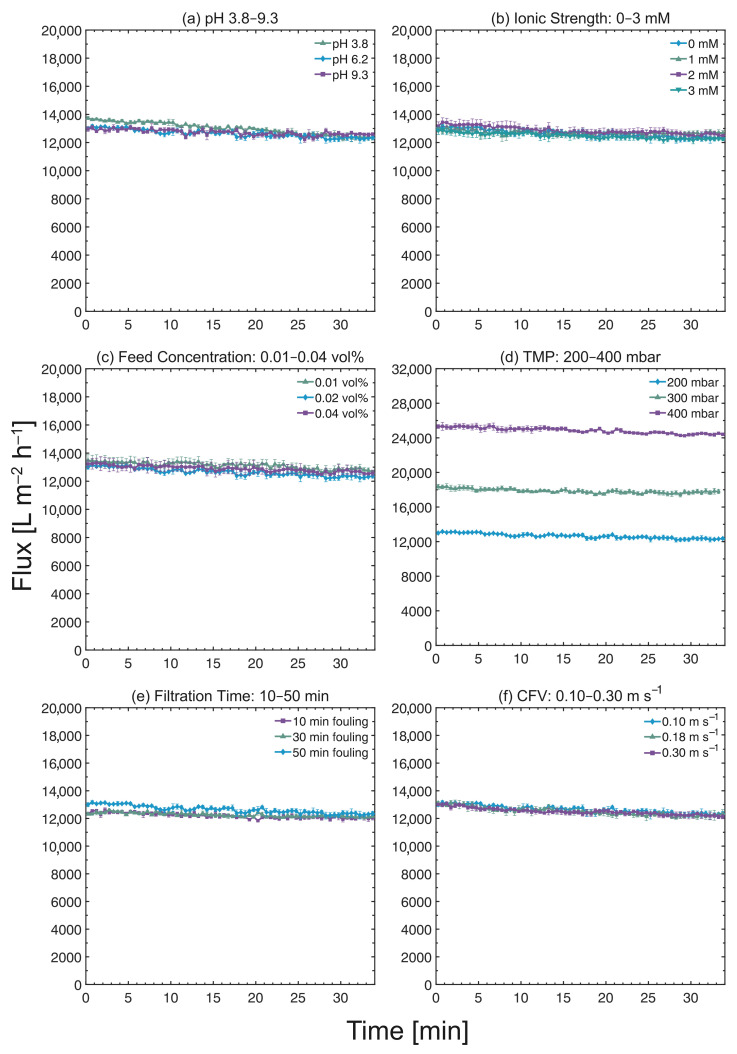
Pure water flux curves prior to cross-flow MF of MCC suspensions, where (**a**) the pH of the MCC suspension was varied from pH 3.8 to 9.3, (**b**) the ionic strength was tested at 0–3 mM, (**c**) the feed concentration range was 0.01–0.04 vol%, (**d**) the TMP range was at 200–400 mbar, (**e**) the filtration times were 10–50 min and (**f**) the CFVs were 0.10–0.30 m s${}^{-1}$. N.B. The flux values were measured for a duration of 30 min, plus several minutes of FDG measurements. Identical conditions for the deionized water were applied in the flux curves in (**c**,**e**). However, different notations were employed to indicate the specific initial flux value it refers to during fouling.

The pure water flux curves of the PES membrane under various feed suspension chemistries and operating conditions are given in Figure A3 in Appendix D. Although the flux values exhibited a consistent linear trend for all conditions, a gradual decline in the pure water flux values was observed. This decline could be attributed to residual foulants from prior experiments, which aligns with findings in earlier studies [21,22]. In Figure A3d, the pure water flux is seen to increase with the operating TMP. The permeabilities of the membrane within the TMP range investigated were calculated by dividing the pure water flux by the TMP. The permeability values remained relatively constant, ranging between 5.9 × 10${}^{4}$ and 6.2 × 10${}^{4}$ L m${}^{-2}$ h${}^{-1}$ bar${}^{-1}$, indicating a minimal influence of TMP on the permeability of the membrane.

**Figure 4:**
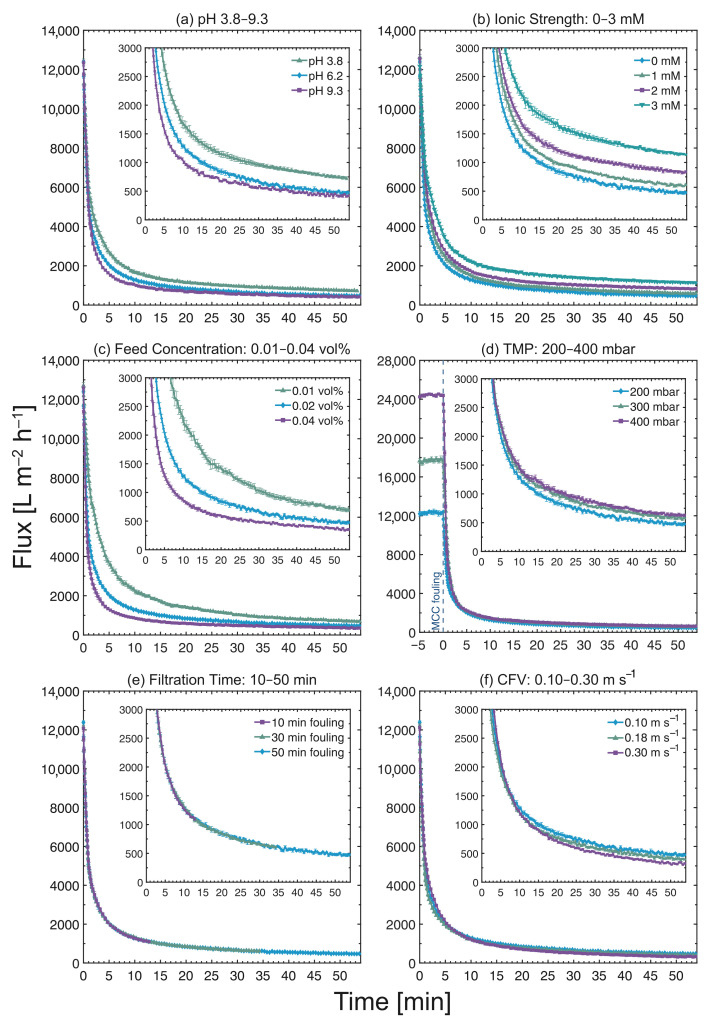
Permeate flux vs. filtration time during the cross-flow MF of MCC suspensions, where (**a**) the pH of the MCC suspension was varied from pH 3.8 to 9.3, (**b**) the ionic strength was tested at 0–3 mM, (**c**) the feed concentration range was 0.01–0.04 vol%, (**d**) the TMP range was at 200–400 mbar, (**e**) the filtration times were 10–50 min and (**f**) the CFVs were 0.10–0.30 m s${}^{-1}$. N.B. The flux curves for pH 6.2 in (**a**), 0 mM in (**b**), 0.02 vol% in (**c**), 200 mbar TMP in (**d**), 50 min fouling in (**e**) and 0.10 m s${}^{-1}$ CFV in (**f**) are identical. The flux values were measured for a duration of 50 min, plus several minutes of FDG measurements.

The permeate flux curves of the cross-flow MF of MCC suspensions are presented in Figure 4. In Figure 4a–c, the pH was tested within the range of pH 4 to 9, the ionic strength was varied by adding 1 mM to 3 mM of NaCl, and the concentration of the feed suspensions ranged from 0.01 to 0.04 vol%, while the cross-flow MF was operated at 200 mbar and 0.10 m s${}^{-1}$ CFV for a duration of 50 min. In Figure 4d–f, the operating TMP range was 200–400 mbar (±5%), the filtration times were 10–50 min, and the CFVs were 0.10–0.30 m s${}^{-1}$ at a feed concentration of 0.02 vol%, pH of 6.2, and no addition of salt. In all cases, the permeate flux declined massively within the first few minutes of MF from initial values of approximately 1.2 × 10${}^{4}$ L m${}^{-2}$ h${}^{-1}$. Given that the nominal particle size of MCC (20 μm) is significantly larger than the nominal pore size of the PES membrane (0.45 μm), and even the smallest particle detected is still above 1 μm based on laser diffraction analysis, the most probable fouling mechanism during the cross-flow MF of MCC is cake fouling caused by the deposition of particles on the membrane surface.

In Figure 4a, the permeate flux dropped to less than 750 L m${}^{-2}$ h${}^{-1}$ at pH 3.8 by the end of MF, while at pHs 6.2 and 9.3, the flux values dropped even further to less than 500 L m${}^{-2}$ h${}^{-1}$. A similar declining trend was also observed by Zhou et al. [21], who found that the permeate flux decline was slower at an acidic pH during the cross-flow MF of MCC operated under transitional/turbulent flow conditions. The slightly slower flux decline at the acidic pH can be attributed to changes in the electrostatic interactions of MCC particles due to variations in their surface charge densities across different pH levels. Under acidic conditions, the surface charges decreased substantially. This effect, combined with the increase in the concentration of particles near the membrane surface, resulted in particle agglomeration, thus shifting the size distribution towards larger sizes. The presence of larger agglomerates reduces the specific surface area in contact with the fluid flow, thereby lowering the specific filtration resistance of the material [31]. Zhou et al. [21] also reported a decrease in the magnitude of the zeta potential of the PES membrane from −87 mV at pH 5.8 to −36 mV at pH 2.6, which indicates reduced electrostatic repulsive forces between the membrane and the MCC particles, thereby promoting particle deposition onto the membrane surface.

In the permeate flux curves at various ionic strengths shown in Figure 4b, significant differences in the flux decline were observed: the final flux values were 477 ± 16, 611 ± 14, 838 ± 22, and 1135 ± 13 L m${}^{-2}$ h${}^{-1}$ for 0 mM, 1 mM, 2 mM, and 3 mM, respectively. The permeate flux decline becomes slower as the ionic concentration is increased. In a study by Lidén et al. [25] on the influence of ionic strength on the filtration behavior of MCC during dead-end filtration, an increase in filtration rate with ionic concentration was reported. As shown in Table 1, the magnitude of the zeta potential decreased upon addition of NaCl. At high ionic concentrations, the repulsive electrostatic forces are reduced owing to the shielding of negative charges, thereby forming larger agglomerates when the concentration of particles increases close to the membrane. This parallels the effect observed with changes in pH, ultimately lowering the specific filtration resistance. In addition, Lidén et al. [25] also reported that the magnitude of the zeta potential of the PES membrane decreased successively upon the addition of more NaCl, thus reducing the electrostatic repulsion between the membrane and the MCC particles at higher ionic concentrations. Another potential effect is that the membrane’s permeability increases with the addition of more ions: larger particles that are deposited on the membrane block its pores less than smaller particles. Similarly, the cake structure and the solidosity (i.e., the volume fraction of solids) in different sections of the cake layer may also vary as a result of altered electrostatic interactions.

Figure 4c shows that the permeate flux decline varied across different feed concentrations: 0.04 vol% exhibited the most severe decline, whilst the lowest decline was observed at 0.01 vol%. At the end of the cross-flow MF, the final flux values were 697 ± 33, 477 ± 16, and 353 ± 21 L m${}^{-2}$ h${}^{-1}$ for 0.01, 0.02, and 0.04 vol%, respectively. At higher concentrations, more particles are present, and there is therefore an increased propensity for MCC particles to deposit onto the membrane surface. A cake layer is formed as a result of particle deposition, which restricts fluid flow through the membrane pores and, ultimately, results in a faster decline in flux. This trend was also observed by Chew et al. [13], where the flux decline increased with suspension concentration in dead-end filtration.

In Figure 4d, the pure water flux values measured five minutes prior to MCC fouling were also included to highlight the difference in initial flux values at different TMPs. The pure water flux values are as expected: when compared to the flux at 200 mbar (∼1.2 × 10${}^{4}$ L m${}^{-2}$ h${}^{-1}$ ), it is roughly 50% higher at 300 mbar (∼1.8 × 10${}^{4}$ L m${}^{-2}$ h${}^{-1}$) and nearly 100% higher at 400 mbar (∼2.4 × 10${}^{4}$ L m${}^{-2}$ h${}^{-1}$). When the cross-flow MF of MCC began, the flux values declined rapidly for all TMPs. By the end of the MF, the flux values had dropped to values below 500, 600, and 650 L m${}^{-2}$ h${}^{-1}$ for TMPs of 200, 300, and 400 mbar, respectively. Consequently, even with varying TMPs (i.e., the driving force for separation), the largest difference in the terminal flux values was only about 30%. This response can be attributed to the faster build-up of a thick fouling layer, as evidenced by the FDG profiles at different TMPs (see Section 3.6 “Influence of TMP”), as well as the formation of compressible cakes [25].

The permeate flux curves for filtration times ranging from 10 to 50 min in Figure 4e followed a consistent trend. All flux curves showed good agreement when superimposed, indicating reproducible MF experiments. It is important to highlight that the flux measurements were terminated after conducting FDG measurements; therefore, the permeate flux values were plotted from when the feed suspension began being circulated up to the specified filtration time plus several minutes of FDG measurements.

The permeate flux curves in Figure 4f show marginal variations in the terminal permeate flux values across different flow regimes. At a CFV of 0.10 m s${}^{-1}$ (*Re*${}_{\mathrm{duct}}$ = 1700), the terminal flux values was below 500 L m${}^{-2}$ h${}^{-1}$, whereas it was below 400 L m${}^{-2}$ h${}^{-1}$ at 0.18 m s^−1^ (*Re*${}_{\mathrm{duct}}$ = 3100), and below 350 L m${}^{-2}$ h${}^{-1}$ at 0.30 m s${}^{-1}$ (*Re*${}_{\mathrm{duct}}$ = 4900). When comparing this trend to the results of the feed chemistry tests, the CFV had minimal influence on the permeate flux. This response was also observed Zhou and Mattsson [22], where the flow regime did not affect the permeate flux during the cross-flow MF of MCC when regenerated cellulose membranes were used.

Based on the permeate flux decline trends found under varying feed and operating conditions, the flux values were influenced, to some extent, by all of the parameters investigated, with the exception of filtration time. Nevertheless, these trends do not provide information on the fouling layer properties or the type of membrane fouling. The sections that follow present the influence of these parameters on the build-up of fouling layers during cross-flow MF.

### 3.3. Influence of pH

**Figure 5:**
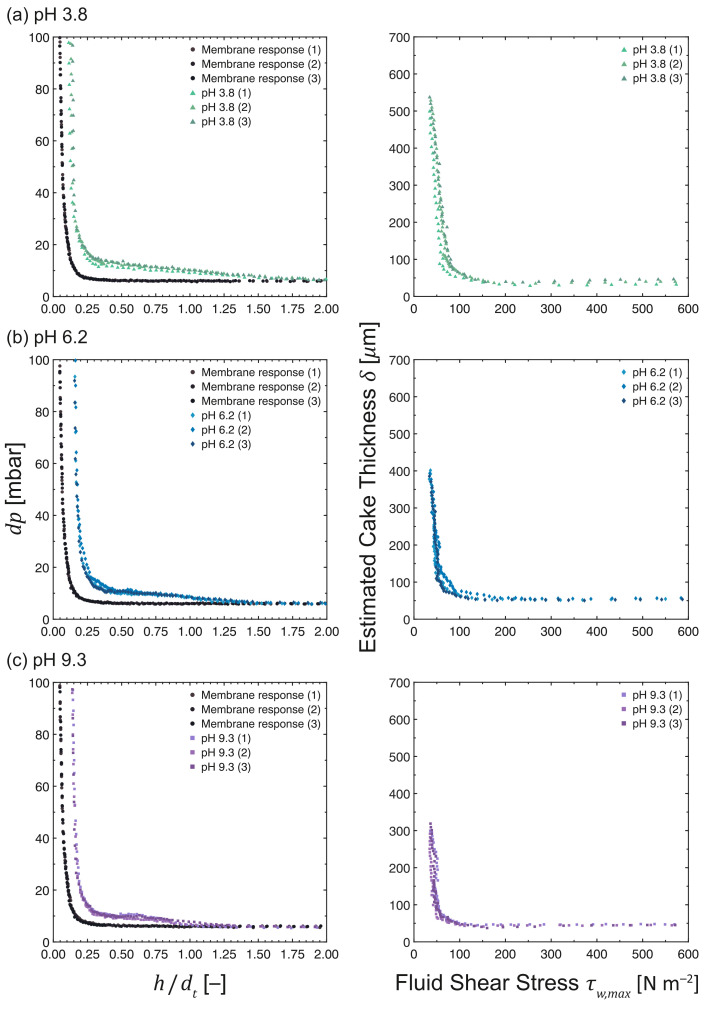
*dp* vs. *h*/*d*${}_{t}$ and δ vs. $\tau_{w,max}$ profiles as measured during the cross-flow MF of MCC suspensions at 0.02 vol%. The pH levels were (**a**) 3.8 (**b**) 6.2 and (**c**) 9.3, while maintaining a constant TMP, filtration time, and CFV at 200 mbar, 50 min, and 0.10 m s${}^{-1}$, respectively. N.B. The *x*-axis of the response of a pristine membrane (•) is *h*${}_{0}$/*d*${}_{t}$ due to the absence of a fouling layer.

The properties of the MCC fouling layers formed during cross-flow MF were investigated using FDG. Figure 5 shows the plots of the differential pressure, *dp*, vs. normalized probe distance, *h*/*d*${}_{t}$, and the cake thickness, δ, vs. fluid shear stress, $\tau_{w,\max}$, profiles during the cross-flow MF of MCC suspensions at 0.02 vol%, where the pH was varied from pH 3.8 to 9.3.

The pristine membrane curves (•) displayed similar responses across all pH levels. Baseline *dp* values were measured at *h*${}_{0}$/*d*${}_{t}$ > 0.25 (asymptotic zone), whereas a sharp increase in *dp* values was observed at *h*${}_{0}$/*d*${}_{t}$ ≤ 0.25 (incremental zone). Comparing the profiles at different pH levels, the response at the acidic pH deviated from the pristine membrane response at *h*/*d*${}_{t}$∼1.75, which is at a normalized probe distance farther than those at close-to-neutral (*h*/*d*${}_{t}$∼1.25) and alkaline (*h*/*d*${}_{t}$∼1.00) conditions. This difference in profiles translates to a variation in the calculated thickness of the fouling layers, where the difference in *h*/*d*${}_{t}$ values between the pristine membrane response and fouling curves corresponds to the fouling layer thickness. At low probe clearing heights (*h*/*d*${}_{t}$ < 0.20), the non-convergence of the fouling and membrane responses indicates the resilience of cake layers formed near the membrane surface.

**Figure 6:**
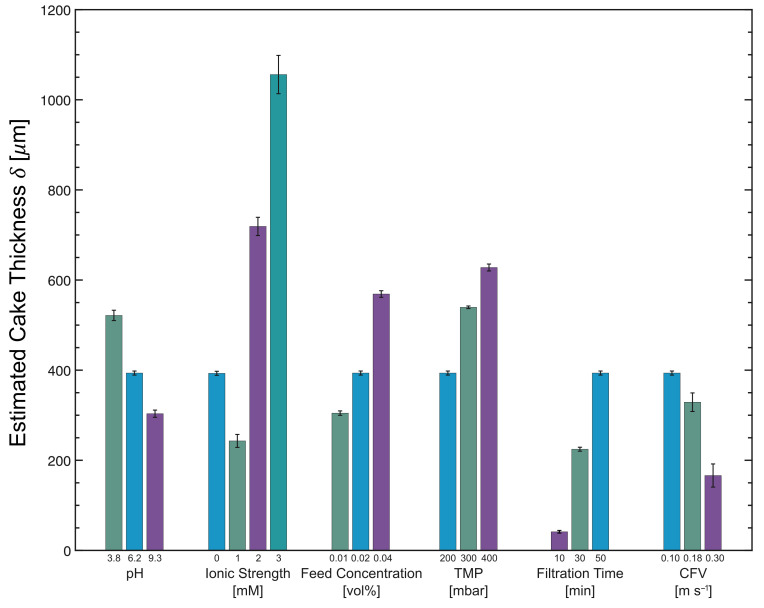
Summary of the fouling layer thickness during cross-flow MF of MCC suspensions under various feed and operating conditions. N.B. An identical dataset was taken for pH 6.2, 0 mM ionic strength, 0.02 vol% feed concentration, 200 mbar TMP, 50 min filtration time, and 0.10 m s${}^{-1}$ CFV.

The δ vs. $\tau_{w,\max}$ profiles in Figure 5 indicate that the fouling layer thickness decreases with increasing pH. The estimated thickness of the fouling layers are 522 ± 12 μm at 35.3 ± 0.8 Pa, 394 ± 5 μm at 34.8 ± 0.6 Pa, and 303 ± 8 μm at 35.6 ± 0.3 Pa for pH 3.8, 6.2, and 9.3, respectively. These values are also depicted in Figure 6, which provides a summary of the fouling layer thickness values under different feed characteristics and operating conditions. At lower pH levels, thicker cake layers were formed, most likely due to the formation of larger agglomerates, and the network between particles was strengthened as a result of weaker repulsive electrostatic forces. In addition, the relatively higher permeate flux promoted the transport of more particles and agglomerates towards the surface of the membrane [32]. On the other hand, the repulsive electrostatic forces between MCC particles and between MCC and the PES membrane at higher pH levels may have facilitated their removal from the fouling layer, along with the shear forces generated by the cross-flow.

From the δ vs. $\tau_{w,\max}$ profiles, the cohesive strength increases in the direction towards the membrane surface. Loose cake layers were readily removed at fluid shear stresses of <100 Pa, with the removal of at least 80% of the cake layer being possible, while thin but resilient cake layers formed close to the membrane surface were observed at higher shear stresses. A plausible explanation for this difference in cohesive strength is the result of a higher local solid pressure being exerted on foulants near the membrane surface and MCC filter cakes exhibiting compressible behavior [26].

The permeabilities of the cake layers at various pH levels were estimated using Equation (3), and the calculated values are given in Table 2. The cake permeability is higher at 2.39 × 10${}^{-12}$ m${}^{2}$ at pH 3.8, whereas they are lower at pHs 6.2 and 9.3 being 1.81 × 10${}^{-12}$ and 1.39 × 10${}^{-12}$ m${}^{2}$, respectively. These values indicate that the fouling layers formed at the acidic pH level are more permeable, despite being thicker than those at close-to-neutral and alkaline conditions.

### 3.4. Influence of Ionic Strength

**Figure 7:**
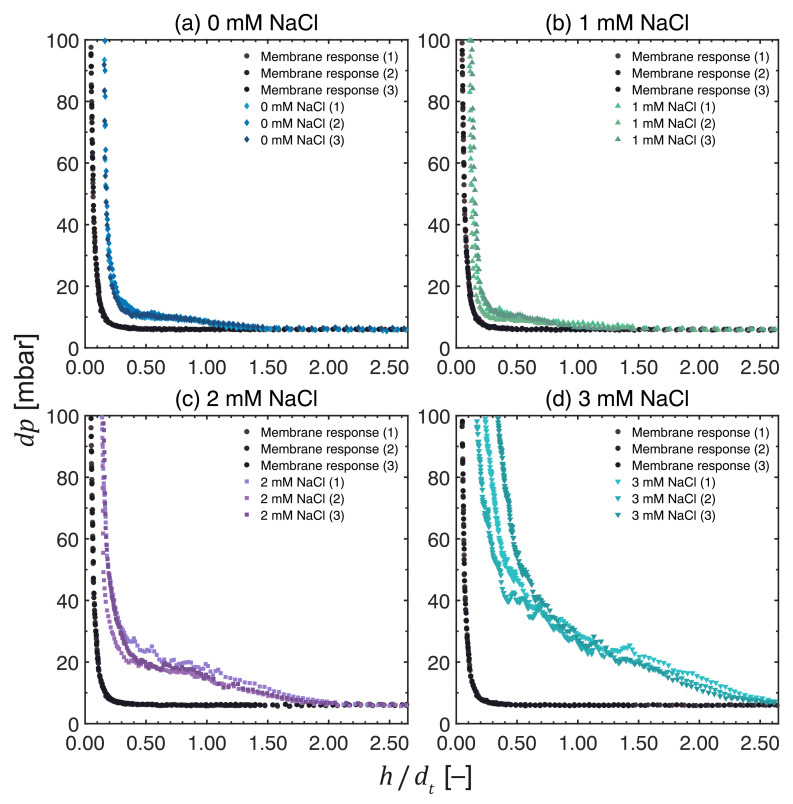
Differential pressure, *dp*, over the FDG nozzle vs. the normalized probe distance, *h*/*d*${}_{t}$, as measured during the cross-flow MF of MCC suspensions at 0.02 vol%. The ionic concentration was tested at (**a**) 0 mM NaCl, (**b**) 1 mM NaCl, (**c**) 2 mM NaCl, and (**d**) 3 mM NaCl, while maintaining a constant TMP, filtration time, and CFV at 200 mbar, 50 min and 0.10 m s${}^{-1}$, respectively. N.B. The *x*-axis of the response of a pristine membrane (•) is *h*${}_{0}$/*d*${}_{t}$ due to the absence of a fouling layer.

The FDG profiles of the fouling layers in Figure 7 show the *dp* vs. *h*/*d*${}_{t}$ plot for the cross-flow MF of MCC suspensions at 0.02 vol%, where the ionic concentration was varied from 0 mM to 3 mM NaCl.

It is evident that there is a significant variation in the pressure profiles at different ionic strengths. The fouling responses deviated more from the pristine membrane curves as the ionic concentration increased, except when 1 mM of NaCl was added, which deviated even less than when no NaCl was added. The exact reason for this behavior is not fully understood; however, consistent results were obtained from triplicate tests, as illustrated in Figure 7b. The trend based on the FDG profiles corresponds to the following thickness of the fouling layer, given from thinnest to thickest: 1 mM, 0 mM, 2 mM, and 3 mM NaCl.

**Figure 8:**
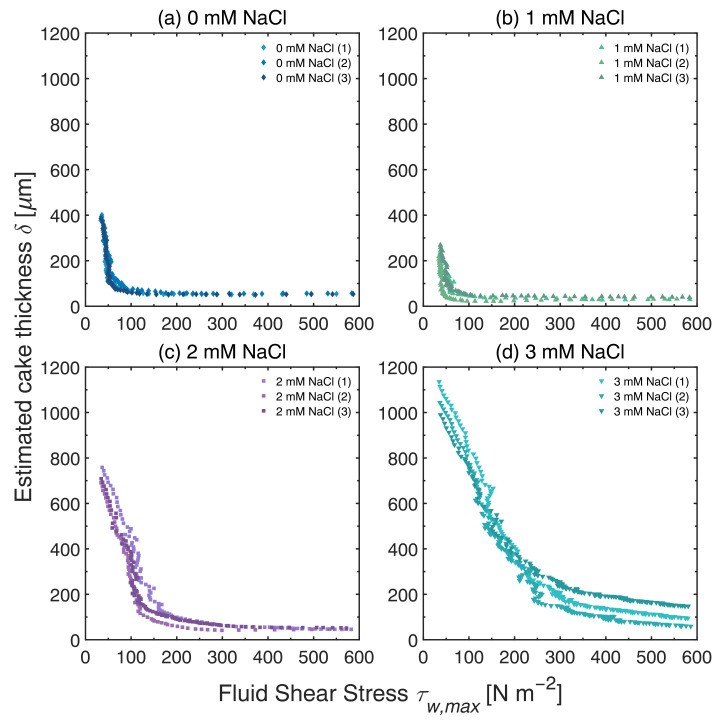
δ vs. $\tau_{w,max}$ profiles during cross-flow MF of MCC suspensions at at 0.02 vol%, with the ionic concentration tested at (**a**) 0 mM NaCl, (**b**) 1 mM NaCl, (**c**) 2 mM NaCl, and (**d**) 3 mM NaCl. The TMP, filtration time, and CFV were kept constant at 200 mbar, 50 min and 0.10 m s${}^{-1}$, respectively.

The thickness vs. shear stress profiles at various ionic concentrations in Figure 8 confirm this trend: the thickest layers were estimated at 3 mM NaCl, while the thinnest layers were measured at 1 mM NaCl. The thickness of the fouling layers in Figure 6 and Figure 8 are 394 ± 5 μm at 34.8 ± 0.6 Pa, 244 ± 14 μm at 36.7 ± 0.7 Pa, 719 ± 20 μm at 35.0 ± 0.7 Pa, and 1057 ± 43 μm at 36.2 ± 0.9 Pa for 0 mM, 1 mM, 2 mM, and 3 mM NaCl, respectively. At higher ionic concentrations, the surface charges are shielded more effectively and larger agglomerates are formed, and their deposition onto the PES membrane surface resulted in thicker cake layers. However, this trend was not observed when 1 mM NaCl was added. As mentioned above, the reason for this behavior is not fully understood, but it may be attributed to the weaker stability of the agglomerates formed at 1 mM NaCl, which promotes their redispersion during cross-flow MF and results in thinner fouling layers being formed. Another possible explanation is related to the change in the surface charge of the membrane, which could also contribute to shielding effects.

As expected, the cohesive strength of the fouling layers increased in the direction towards the membrane surface. It is, however, interesting to compare the shape of the profiles of pH 3.8 in Figure 5a with those of 2 and 3 mM NaCl in Figure 8c,d. It is evident that the properties of the fouling layers differ: the thicknesses of the cake layers at 2 and 3 mM NaCl are much greater than those at pH 3.8. The profiles of cake thickness vs. shear stress vary too. At pH 3.8, the gradient in the lower shear stress region is very steep, whereas the corresponding gradients for 2 and 3 mM NaCl plots are much less steep. Furthermore, the permeate flux values at pH 3.8 are lower than those calculated at 2 and 3 mM NaCl. These observations suggest that the fouling layers formed after adding 2 and 3 mM NaCl are thicker and more resilient but have a higher permeability. Despite detecting a slight variation in surface charges among these cases based on zeta potentials, it may nevertheless be insufficient to explain the differences observed, although one evident difference is observed: at pH 3.8, the surface charge falls below most p*K*${}_{a}$ values for organic acids within these systems. This means that the reduction in surface charges from pH 6.2 to 3.8 implies that point charges are cancelled out. However, as the surface charge decreases due to an increase in ionic strength, the point charges in the form of acids are only weakened but not cancelled out entirely. Thus, one possible explanation for this is that different interactions take place between particles in the fouling layer and these, in turn, give rise to variations in the properties of the fouling layer.

Comparing the profiles at various ionic concentrations, the shear stress required to remove fouling layers down to a given cake thickness increases with ionic strength, with the exception of 1 mM NaCl. This indicates that, in thicker cakes, stronger initial cake layers are formed close to the membrane surface due to compressive forces, and removing these layers require more shear. This observation is supported by the study of Lidén et al. [25], who noted variations in the hydrostatic pressure at different cake heights in the presence of ions during dead-end filtration, forming moderately to highly compressible filter cakes.

Regarding the repeatability and reproducibility of the cross-flow MF experiments, the uncertainty values of the fouling layer thickness increased with ionic strength. At high ionic strengths, the packing of the fouling layers may be less structured, and their packing densities could vary locally due to weaker repulsive electrostatic forces. This variation could result in a heterogeneous distribution of foulants on the membrane surface [33]. Given that FDG measurements were made at a specific location within the filtration cell, i.e., at the center of the active membrane area, the variation in deposition of foulants in that region could contribute to a larger variation in the estimated thickness.

### 3.5. Influence of Feed Concentration

**Figure 9:**
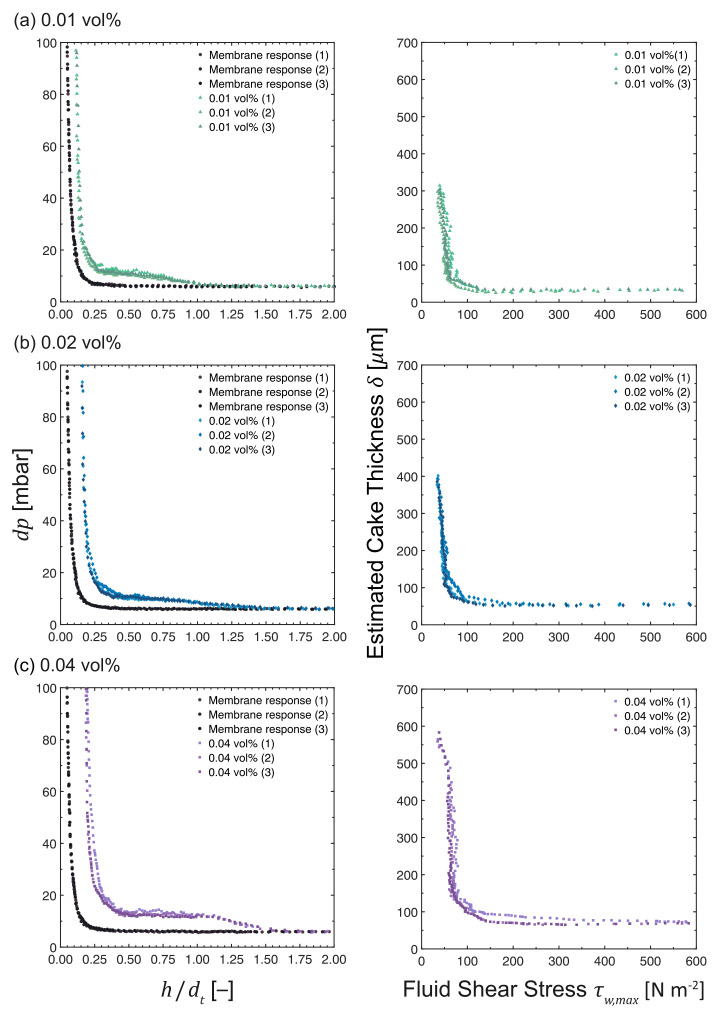
*dp* vs. *h*/*d*${}_{t}$ and δ vs. $\tau_{w,max}$ profiles as measured during the cross-flow MF of MCC suspensions at an unadjusted pH of 6.2. The feed concentration was varied at (**a**) 0.01 vol%, (**b**) 0.02 vol% and (**c**) 0.04 vol%, while maintaining a constant TMP, filtration time, and CFV at 200 mbar, 50 min and 0.10 m s${}^{-1}$, respectively. N.B. The *x*-axis of the response of a pristine membrane (•) is *h*${}_{0}$/*d*${}_{t}$ due to the absence of a fouling layer.

The *dp* vs. *h*/*d*${}_{t}$ and δ vs. $\tau_{w,\max}$ profiles of feed concentrations ranging from 0.01 to 0.04 vol% are presented in Figure 9. The fouling responses deviated more from the pristine membrane curves as the feed concentration increased. The response at 0.01 vol% began to deviate at *h*/*d*${}_{t}$∼1.00, which is at a probe distance closer than those at 0.02 vol% and 0.04 vol%, where deviations started at *h*/*d*${}_{t}$∼1.25 and *h*/*d*${}_{t}$∼1.50, respectively. This trend implies that the thinnest fouling layer is estimated at 0.01 vol%, whereas the thickest fouling layer is measured at 0.04 vol% despite having the most severe flux decline, as observed in Figure 4c.

This trend was confirmed by plotting the cake thickness vs. shear stress at the investigated feed concentration range. The thicknesses of the fouling layers were 305 ± 5 μm at 37.6 ± 1.4 Pa, 394 ± 5 μm at 34.8 ± 0.6 Pa, and 569 ± 7 μm at 36.3 ± 1.0 Pa for 0.01, 0.02, and 0.04 vol%, respectively. At higher feed concentrations, the build-up of the fouling layer will be faster due to a higher particle concentration, which results in more particles being transported towards the surface. In all cases, the upper parts of the cake layer were easily removed. Although the thickness of the more resilient layer increased with increasing feed concentration, the form of the plots is very similar. This behavior indicates that not only the build-up of the fouling layer but also the interactions between particles remain unchanged within the concentration range investigated.

### 3.6. Influence of Transmembrane Pressure

**Figure 10:**
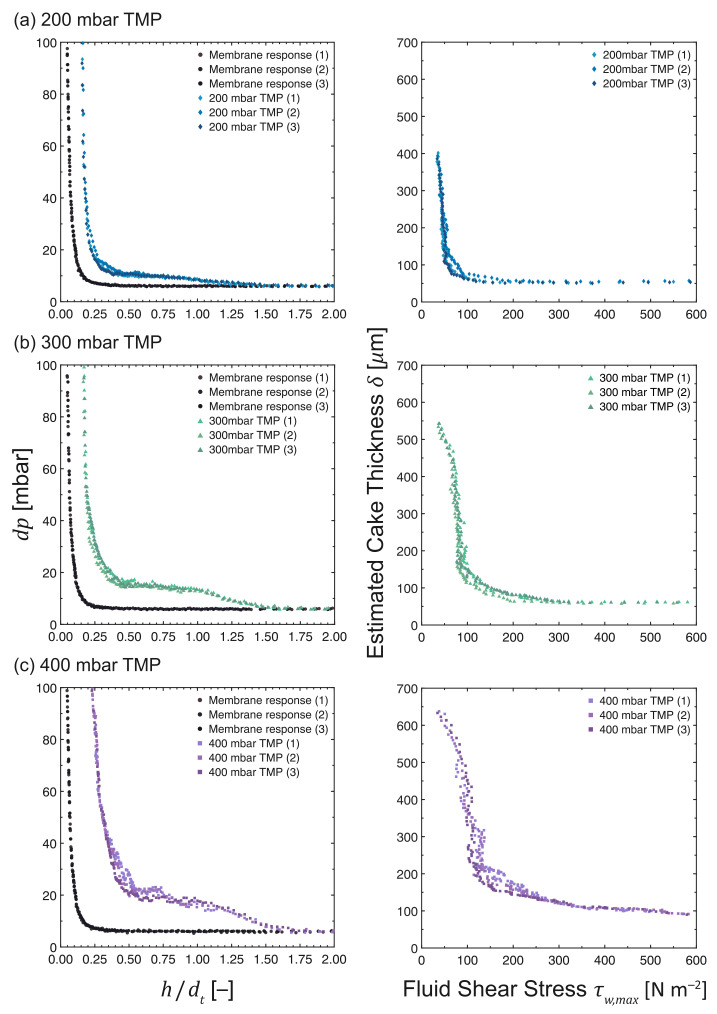
*dp* vs. *h*/*d*${}_{t}$ and δ vs. $\tau_{w,max}$ profiles as measured during the cross-flow MF of MCC suspensions at 0.02 vol%. The operating TMP was varied at (**a**) 200 mbar, (**b**) 300 mbar and (**c**) 400 mbar, while maintaining a constant filtration time and CFV at 50 min and 0.10 m s${}^{-1}$, respectively. N.B. The *x*-axis of the response of a pristine membrane (•) is *h*${}_{0}$/*d*${}_{t}$ due to the absence of a fouling layer.

Figure 10 presents the FDG profiles for the cross-flow MF of MCC suspensions at a concentration of 0.02 vol%, where the TMP was varied from 200 mbar to 400 mbar. The pristine membrane curves at different TMPs showed similar responses, whereas the deviation of the fouling responses from the pristine membrane increased with TMP. The response at a TMP of 200 mbar started to deviate at *h*/*d*${}_{t}$∼1.25. At higher TMPs, the deviation began at a much farther distance: it was at *h*/*d*${}_{t}$∼1.50 for 300 mbar and at *h*/*d*${}_{t}$∼1.75 for 400 mbar. This difference in responses would correspond to an order in fouling layer thickness, starting with the thinnest layer at 200 mbar, followed by 300 mbar and ending with the thickest layer at 400 mbar.

The thickness vs. shear stress profiles at different TMPs in Figure 10 show that the estimated fouling layer thicknesses are 394 ± 5 μm at 34.8 ± 0.6 Pa, 540 ± 3 μm at 37.4 ± 0.6 Pa, and 628 ± 8 μm at 38.6 ± 2.4 Pa for 200 mbar, 300 mbar, and 400 mbar, respectively. Thicker fouling layers were formed when the cross-flow MF was operated at higher TMPs, an observation that was also reported by Jones et al. and Lister et al. for ballotini suspensions [14,15]. Since higher permeate flux values were observed at higher TMPs during the initial phase of the MF (Figure 4d), more particles were transported towards the membrane surface. However, in the latter stages of the MF, the difference in flux values was less significant as the cake layer built up.

Despite exhibiting a large variation in initial flux, the terminal permeate flux values were relatively close: all values were less than 4% of the initial pure water flux. This trend in flux decline suggests certain similarities in fouling behavior across different TMPs. The cohesive strength of the fouling layers formed at different TMPs also followed the same trend as the other parameters: cohesive strength increased in the direction towards the membrane surface. The cake layers close to the membrane surface experienced a higher solid compressive pressure, making them denser, stronger, and more resistant to the applied fluid shear stress [21]. These dense cake structures have a high solidosity and filtration resistance, leading to a significant decline in permeate flux. The cohesive strength of the cake layers also increased with increasing TMP. It is evident from Figure 10 that an increase in TMP requires more fluid shear stress to remove cake layers at the same distance from the membrane surface.

### 3.7. Influence of Filtration Time

**Figure 11:**
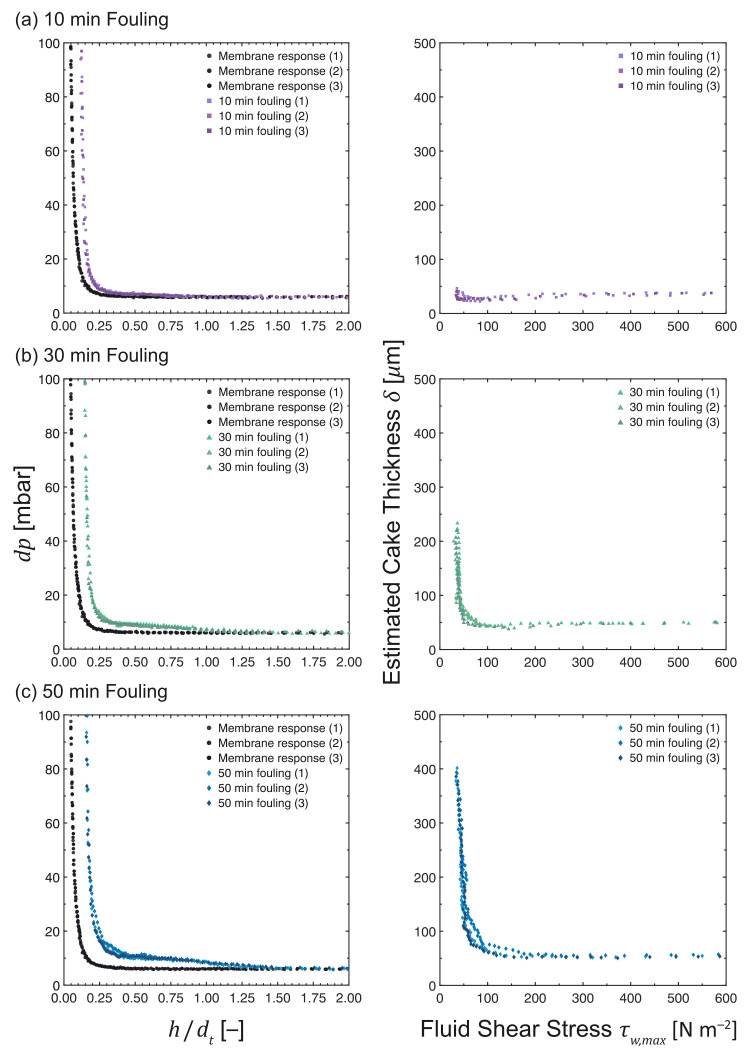
*dp* vs. *h*/*d*${}_{t}$ and δ vs. $\tau_{w,max}$ profiles as measured during the cross-flow MF of MCC suspensions at 0.02 vol%. The filtration time was varied at (**a**) 10 min, (**b**) 30 min and (**c**) 50 min, while maintaining a constant TMP and CFV at 200 mbar and 0.10 m s${}^{-1}$, respectively. N.B. The *x*-axis of the response of a pristine membrane (•) is *h*${}_{0}$/*d*${}_{t}$ due to the absence of a fouling layer.

The *dp* vs. *h*/*d*${}_{t}$ and δ vs. $\tau_{w,\max}$ profiles of the cross-flow MF of MCC suspensions, with filtration times ranging from 10 to 50 min, are given in Figure 11. The deviation of the fouling responses from the pristine membrane curve increased with filtration time. The least deviation was observed at 10 min, while the most significant deviation was monitored at 50 min: this trend translates to a thin fouling layer being formed after 10 min of MF, which increases in thickness as the filtration time is extended.

The estimated fouling layer thicknesses in Figure 11 are 41 ± 3 μm at 36.4 ± 0.7 Pa, 225 ± 4 μm at 36.0 ± 0.5 Pa, and 394 ± 5 μm at 34.8 ± 0.6 Pa for 10, 30, and 50 min of cross-flow MF, respectively. The thickness of the fouling layer increases with filtration time. At the shortest filtration time of 10 min, very thin cake layers were measured since the fouling layer had just started to build up. It is interesting to note that this layer is quite resilient, with a cake thickness that is independent of the applied fluid shear. As the filtration time is extended, the fouling layer gradually becomes thicker. The thickness of the resilient layer increased slightly after 30 min, accompanied by a much thicker layer of less resilient deposits. After 50 min, the resilient layer had nearly the same thickness as the 30-min MF, but the thickness of the looser layer increased substantially.

The cohesive strength of the fouling layers also increased in the direction towards the membrane surface, with longer filtration times requiring more shear stress to remove cake layers down to a given thickness compared to those at shorter filtration times. At an applied shear stress of 50 Pa, the remaining cake thicknesses were 28, 64, and 181 μm for filtration times of 10, 30, and 50 min, respectively. Upon increasing the applied shear stress to 100 Pa, the thicknesses of the remaining cake decreased to 26, 48, and 66 μm.

### 3.8. Influence of Cross-Flow Velocity

**Figure 12:**
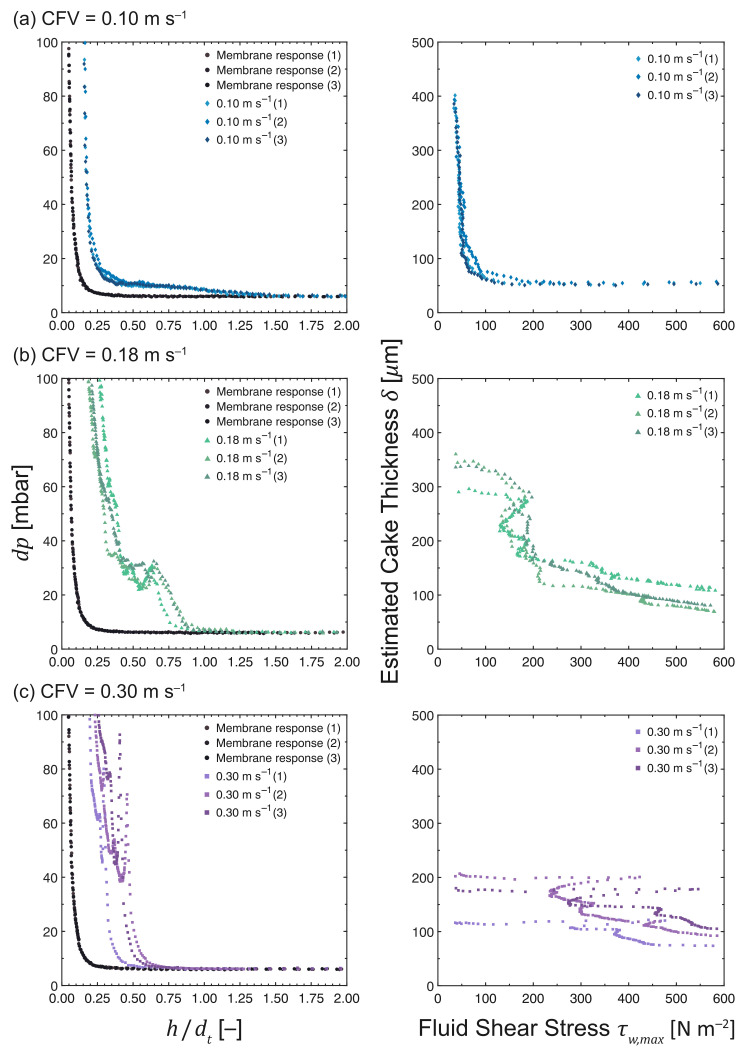
*dp* vs. *h*/*d*${}_{t}$ and δ vs. $\tau_{w,\max}$ profiles as measured during the cross-flow MF of MCC suspensions at 0.02 vol%. The CFV was varied at (**a**) 0.10 m s${}^{-1}$ (*Re*${}_{\mathrm{duct}}$ = 1700), (**b**) 0.18 m s${}^{-1}$ (*Re*${}_{\mathrm{duct}}$ = 3100) and (**c**) 0.30 m s${}^{-1}$ (*Re*${}_{\mathrm{duct}}$ = 4900), while maintaining a constant TMP and filtration time at 200 mbar and 50 min, respectively. N.B. The *x*-axis of the response of a pristine membrane (•) is *h*${}_{0}$/*d*${}_{t}$ due to the absence of a fouling layer.

The FDG profiles at CFVs ranging from 0.10 to 0.30 m s${}^{-1}$ are shown in Figure 12. Significant variation in fouling behavior was observed at different CFVs despite marginal differences in flux decline, as can be seen in Figure 4f. In the laminar regime at a CFV of 0.10 m s${}^{-1}$ (*Re*${}_{\mathrm{duct}}$ = 1700), the fouling responses were reproducible, exhibiting minimal variations between experiments. When the MF was operated in the transitional regime at a CFV of 0.18 m s${}^{-1}$ (*Re*${}_{\mathrm{duct}}$ = 3100), the variation between experiments and its deviation from the pristine membrane curve increased. A significantly sharper rise in *dp* was observed in the region where *h* /*d*${}_{t}$is between 0.75 and 1.00, followed by a slight drop in *dp* at *h*/*d*${}_{t}$ = 0.5–0.75. At high *dp* values, a substantial difference was observed between the fouling and the pristine membrane responses. The variation between experiments was considerably more significant when the MF was operated in the turbulent regime at a CFV of 0.30 m s${}^{-1}$ (*Re*${}_{\mathrm{duct}}$ = 4900). A much steeper surge in *dp* was observed at a relatively constant *h*/*d*${}_{t}$, which eventually collapsed as *h*/*d*${}_{t}$ decreased, followed by a couple of steep rises in *dp* until it reached 100 mbar. Under transition or turbulent conditions, foulant deposition may vary at different locations within the flow cell; consequently, the flow conditions will not only vary at different locations but also be dependent on the design of the filtration equipment.

The δ vs. $\tau_{w,\max}$ profiles in Figure 12 show that the estimated fouling layer thicknesses are 394 ± 5 μm at 34.8 ± 0.6 Pa, 329 ± 21 μm at 39.4 ± 2.0 Pa, and 166 ± 26 μm at 37.0 ± 0.4 Pa for 0.10 m s${}^{-1}$ (*Re*${}_{\mathrm{duct}}$ = 1700), 0.18 m s${}^{-1}$ (*Re*${}_{\mathrm{duct}}$ = 3100), and 0.30 m s${}^{-1}$ (*Re*${}_{\mathrm{duct}}$ = 4900), respectively. This trend indicates that the thickness of the fouling layer decreases as the CFV increases, which concurs with the findings of Jones et al. [14]. Under high cross-flow conditions, less material was deposited on the membrane surface. Thinner cake layers formed at higher CFVs have higher hydraulic resistances than thicker ones formed at lower CFVs [34]. The deposition is also expected to be inhomogeneous, as a high variation in fouling behavior was observed in the transitional and turbulent flow regimes.

Regarding cohesive strength, the highest CFV had the strongest and most resilient layers. In the laminar regime, the cake layers were sheared off continuously, and a slight increase in the applied shear stress (∼60 Pa) was necessary to remove 80% of the fouling layer, considering that a large part of the cake is a loose polarization layer. The fouling layers formed under both transition and turbulent conditions can withstand the shear forces initially, and their removal required much higher fluid shear. The layers formed in the transitional regime were detached in larger pieces, as indicated by the small drop in *dp* at *h*/*d*${}_{t}$ = 0.5–0.75 in Figure 12b. This removal occurred at a fluid shear stress that was sufficient to detach this portion of the cake layer and was followed by a gradual removal of inner cake layers. When compared to the cake layers formed under laminar conditions, thinner cakes exhibiting at least two regions in terms of resilience were observed in the transitional flow regime. Under turbulent conditions, thin yet highly resilient cake layers were formed that were barely affected by the shear forces. The thickness of the cake layers remained constant initially, despite increasing the applied fluid shear. Subsequently, the top portion of the cake layers was removed under significantly higher shear stress.

The CFV is a crucial operational parameter in controlling fouling during cross-flow filtration. It can influence the velocity of the particles of the feed stream as well as the packing of the cake layers formed [34]. In cases where comparable membrane performances are observed based on permeate flux, cleaning protocols should be customized in accordance with the mechanical and chemical properties of fouling layers.

## 4. Concluding Remarks

This study highlights fluid dynamic gauging (FDG) as a technique for monitoring the fouling behavior of cellulosic materials in situ during membrane filtration. The FDG results obtained demonstrate that the thickness and cohesive strength of fouling layers formed during the cross-flow microfiltration (MF) of microcrystalline cellulose (MCC) are markedly influenced by the feed suspension chemistry and operating conditions. The thickness of the cake layers formed varies, depending on the changes made to the feed characteristics and the operating conditions. The cohesive strength of the fouling layers increases in the direction towards the membrane surface, with loose layers forming at the upper portion of the cake while resilient layers form near the membrane surface.

The characteristics of fouling layers were found to be influenced by pH and ionic strength, most likely due to variations in particle–particle and particle–membrane interactions under different conditions. At pH 3.8 and ionic strengths of 2 and 3 mM, thicker and more permeable cake layers were formed, possibly due to the presence of larger agglomerates resulting from weaker repulsive electrostatic forces. The reduction in surface charges at an acidic pH level suggests that the acid-based point charges are cancelled out, while at higher ionic concentrations, they are rather weakened but not cancelled out. Higher feed concentrations led to faster build-up of the fouling layers, resulting in thicker and more resilient cake layers. However, the interactions between particles remain unchanged within the concentration range investigated. The cohesive strength of the cake layers increased with transmembrane pressure (TMP), increasing the fluid shear stress required to remove cake layers at the same distance from the membrane surface. Very thin yet resilient cake layers were observed after the 10 min cross-flow MF, and their resilience was unaffected by the applied fluid shear. At longer filtration times, the height of the cake layers increased, accompanied by a substantial increase in the thickness of looser cake layers. The cross-flow velocity (CFV) influenced the thickness and resilience of fouling layers significantly, resulting in thinner yet stronger cake layers under high CFVs, which required much higher fluid shear for removal. In the transitional and turbulent flow regimes, a considerable variation in fouling behavior was observed, likely attributed to the inhomogeneous deposition of MCC particles.

Electrostatic interactions, particle count and size distribution, solid compressive pressures, and shear forces due to cross-flow are crucial factors that must be taken into consideration when developing operational procedures and cleaning protocols in pressure-driven membrane processes. These factors can modify, individually or collectively, the mechanical and chemical properties of fouling layers. Future research prospects may include exploring the interactions of these factors, along with employing advanced surface characterization techniques and molecular dynamics simulations. These analyses could provide deeper insights into the fouling behavior of cellulosic materials. Nevertheless, this comprehensive study on the influence of the feed suspension chemistry and operating conditions takes a step towards employing improved operating and cleaning strategies founded on the properties of fouling layers. This is particularly important when similar membrane performances are observed based on the decline in permeate flux.

## Figures and Tables

**Table 1:** Size distributions and zeta potentials of the feed MCC suspensions at 0.02 vol% at varying pH levels and ionic strengths.

Feed Condition	D${}_{10}$ [μm] ^1^	D${}_{50}$ [μm] ^1^	D${}_{90}$ [μm] ^1^	Zeta Potential [mV]
pH 4	8.8 ± 0.1	21.7 ± 0.2	45.9 ± 0.5	−19.7 ± 2.2
pH 6	8.7 ± 0.04	21.7 ± 0.1	45.6 ± 0.3	−33.4 ± 2.5
pH 9	8.7 ± 0.04	21.6 ± 0.1	45.6 ± 0.3	−34.3 ± 3.0
1 mM NaCl	8.8 ± 0.02	21.7 ± 0.1	45.7 ± 0.2	−17.0 ± 2.6
2 mM NaCl	8.8 ± 0.01	21.8 ± 0.02	46.0 ± 0.2	−14.9 ± 2.4
3 mM NaCl	8.8 ± 0.01	21.8 ± 0.03	46.1 ± 0.1	−13.5 ± 2.1

^1^ D${}_{x}$ indicates the size below which x% of the material is contained.

**Table 2:** Permeability values of the cake layer at various pH levels.

pH of Feed Suspension	*k* [m${}^{2}$]
pH 3.8	(2.39 ± 0.06) × 10${}^{-12}$
pH 6.2	(1.81 ± 0.03) × 10${}^{-12}$
pH 9.3	(1.39 ± 0.05) × 10${}^{-12}$

## Data Availability

The data presented in this study are available on request from the corresponding author.

## Abbreviations

The following abbreviations are used in this manuscript:

CFV	Cross-flow velocity [m s${}^{-1}$]
FBRM	Focused Beam Reflectance Measurement
FDG	Fluid dynamic gauging
MCC	Microcrystalline cellulose
MF	Microfiltration
NF	Nanofiltration
PES	Polyethersulfone
RO	Reverse osmosis
TMP	Transmembrane pressure
UF	Ultrafiltration

## Appendix A. Particle Size Distribution

**Table A1:** Size distributions of the feed MCC suspensions at 0.15 vol% under varying pH and ionic strength.

Feed Condition	D${}_{10}$ [μm] ^1^	D${}_{50}$ [μm] ^1^	D${}_{90}$ [μm] ^1^
pH 4	8.8 ± 0.1	21.7 ± 0.1	45.8 ± 0.5
pH 6	8.8 ± 0.1	21.8 ± 0.1	45.7 ± 0.2
pH 9	8.7 ± 0.02	21.6 ± 0.02	45.7 ± 0.2
1 mM NaCl	8.7 ± 0.02	21.6 ± 0.03	45.3 ± 0.1
2 mM NaCl	8.8 ± 0.05	21.8 ± 0.1	45.8 ± 0.2
3 mM NaCl	8.8 ± 0.02	21.7 ± 0.03	45.9 ± 0.1

^1^ D${}_{x}$ indicates the size below which x% of the material is contained.

## Appendix C. Zeta Potential of MCC Particles

**Table A2:** Zeta potentials of MCC particles in the feed suspensions at varying feed concentration.

Feed Concentration [vol%]	Zeta Potential [mV]
0.01	−35.6 ± 3.1
0.04	−36.8 ± 2.9
0.15	−36.3 ± 3.0

## Appendix D. Pure Water Flux Profiles

The pure water flux profiles of the PES membranes at different feed suspension chemistries and operating conditions are shown in Figure A3.
